# First report and genetic diversity of porcine bufavirus in China

**DOI:** 10.1186/s12985-019-1278-6

**Published:** 2020-01-06

**Authors:** Yan-Kuo Sun, Yong-Jie Chen, Yu Cai, Di-Hua Zhu, Hao-Ming Pan, Ying-Fang Wei, Xiao-Liang Han, Chi-Hai Ji, Gang Lu, Heng Wang, Chun-Quan Ma, Gui-Hong Zhang

**Affiliations:** 1 College of Veterinary Medicine, South China Agricultural University, Guangzhou, Guangdong Province China; 2 College of Veterinary and National Engineering Research Center for Breeding Swine Industry, South China Agricultural University, Guangzhou, 510642 China; 3 Department of Animal Medicine, Foshan University, Foshan, Guangdong Province China

**Keywords:** Porcine bufavirus, China, Prevalence, Phylogenetic analysis, Homology analysis

## Abstract

**Background:**

Bufavirus is a newly discovered zoonotic virus reported in numerous mammals and humans. However, the epidemiological and genetic characteristics of porcine bufaviruses (PBuVs) in China remain unclear.

**Methods:**

To detect PBuVs in China, 384 samples (92 fecal and 292 serum specimens) were collected from 2017 to 2018, covering six provinces in China, and were evaluated by nested PCR. Further, the positive samples from different provinces were selected to obtain the complete genome of Chinese PBuVs.

**Results:**

The prevalence rate of PBuV was 16.7% in Chinese domestic pigs in the Guangdong, Guangxi, Fujian, Jiangxi, Anhui, and Henan provinces. Additionally, the positive rate of fecal specimens was higher than that of the serum samples. Next, we sequenced nine near-complete genomes of Chinese field PBuV strains from different provinces. Homology and phylogenetic analyses indicated that Chinese PBuVs have high genetic variation (93.3–99.2%), showed higher nucleotide identity with an Austrian PBuV strain (KU867071.1), and developed into different branches within the same cluster.

**Conclusion:**

To our knowledge, this is the first report on PBuV in China, expanding the geographic boundaries of PBuV circulation. Our data demonstrate that PBuVs are widely distributed in the six Chinese provinces. Moreover, these Chinese PBuVs exhibit genetic variation and continuous evolution characteristics. Taken together, our findings provide a foundation for future studies on bufaviruses.

## Background

Taxonomically, porcine bufavirus (PBuV) belongs to the genus *Protoparvovirus* and subfamily *Parvovirinae.* It is a small and non-enveloped virus with a non-segmented, single-stranded, 4–6 kb DNA genome [1, 2], which encodes non-structural protein 1 (NS1), a putative structural protein 1 (VP1), small hypothetical protein, and structural protein 2 (VP2) [3–9]. Bufavirus has been detected in humans, non-human primates, bats, canines, and rats [4–10]. In 2016, PBuV was first identified in fecal samples of domestic pigs in Hungary by viral metagenomics and polymerase chain reaction (PCR) methods. Its genome is genetically distinct from those of human and other mammalian-borne bufaviruses. It is also known that this virus is highly prevalent in domestic pigs and closely related to posterior paraplegia. Furthermore, another PBuV was shortly detected indiarrheic and normal fecal samples from piglets in Austria. This study revealed that the Austrian strains exhibited 93% genetic diversity to the first identified PBuV strain and the PBuV prevalence was comparatively lower in the investigated farms. However, the distribution of PBuV in the global pig population remains to be determined. To date, there have been only two reports regarding PBuV [2, 8]. Hence, the epidemic and molecular knowledge of PBuV is limited in China.

Infection by bufavirus has not been clearly associated with its pathogenicity. However, since the first report of bufavirus in the fecal specimen from a child with diarrhea in 2012, the viruses have been detected in numerous diarrheal cases in humans [1, 9, 11, 12]. Bufavirus from other species has rarely been reported as pathogenic [5–7, 10]. In a previous report, PBuV showed a higher detection rate in pigs with posterior paraplegia than that in healthy pigs; however, direct evidence and knowledge on epidemiology of this virus are limited [2]. Although the virus has been detected in diarrheic fecal samples of pigs, its relationship with diarrhea remains unclear [2]. Given the close interaction between humans and pigs in daily life, additional epidemiological studies are needed.

In this study, PBuV DNA was identified in both serum and fecal samples collected from clinical healthy Chinese pigs and further characterized by sequencing. Nine full length PBuV sequences were determined. To the best of our knowledge, our study is the first to describe PBuV in domestic Chinese pigs.

## Methods

### Sample collection

From December 2017 to November 2018, 292 serum and 92 fecal samples from healthy pigs (without obvious clinical symptoms), respectively, were collected from 112 commercial pig farms in six provinces (Guangdong, Guangxi, Jiangxi, Fujian, Henan, and Anhui). Samples were collected under the animal ethics guidelines and approved by the Animal Care and Use Committee of South China Agriculture University (Issue Number: 2017–07). The samples were stored at − 80 °C immediately after collection.

### Sample processing and viral DNA extraction

The fecal and serum samples were suspended at a proportion of 10% (wt/vol) in Dulbecco’s modified Eagle medium. The mixture was centrifuged at 8000×*g* at 4 °C for 20 min, and the supernatant was collected. Viral DNA was extracted using the TIANamp Virus DNA/RNA Kit (TianGen, Beijing, China) according to the manufacturer’s instructions and then stored at − 80 °C until analysis.

### Development of a nested-PCR screening to investigate the prevalence of PBuV

Initially available nucleotide sequences of bufavirus were downloaded from a public database (NCBI). All bufavirus sequences from multiple species were aligned using Clustal W (Mega 6) and nested-PCR primers were designed based on these regions. To investigate the prevalence of this bufavirus in China, nested PCR primers were designed according to the conservative regions in the complete genome based on the reference PBuV strains (GenBank accession number: KU867071 and KT96507) (Table 1). The PCR was performed in 96-well plates in a 25-μL reaction volume containing 5 μL of DNA, 12.5 μL of Multiplex PCR Master Mix (Vazyme Biotech, Nanjing, China), 0.5 μL of 0.2 mM each primer, 4 μL of distilled water, and 2.5 μL of template of the samples. The product of the first-round PCR was used as the template for the second round of PCR. Both rounds of PCR were performed under the following conditions: 5 min at 95 °C, followed by 25 cycles for 1 min at 95 °C, 30 s at 56 °C, and 1 min at 72 °C, and a final extension step at 72 °C for 5 min. The PCR products were purified using the AxyPrep DNA gel extraction kit (Axygen, USA) t and sequenced using the Sanger method (BGI, Guangzhou, China).

**Table 1:** Primers used in this study

Fragment	Sequence of PCR primers	Position in genome^a^	Length of PCR products^a^
A^a^	5′-TGACTATACTCTGGACATTAAC − 3′	1–22	1303 bp
	5′-CATKATTGGTTGTCTGTGTTC − 3′	1283–1303	
B^a^	5′-CCATGCAATCATGTGCTGC −3′	916–934	1380 bp
	5′-AGTTTGTTGTATTCCAAATCGT −3′	2274–2295	
C^a^	5′-TGGATACAACTATCTCGGACC −3′	2194–2214	1996 bp
	5′-TATGTCTGGAAGGTTGTAGGT −3′	4169–4189	
nPBuV-1F^b^	5′-CCATGCAATCATGTGCTGC −3′	916–934	1380 bp
nPBuV-1R^b^	5′-AGTTTGTTGTATTCCAAATCGT −3′	2274–2295	
nPBuV-2F^b^	5′-AAGAAGCAGGCAACCTAGG −3′	1110–1128	194 bp
nPBuV-2R^b^	5′-CATKATTGGTTGTCTGTGTTC −3′	1283–1303	

^a^PCR primers of the near-complete genome, position in the genome, and length of PCR products with respect to the porcine bufavirus strain 61 (accession no. KU867071) and genome

^b^Nested PCR primers of porcine bufavirus

### Full–length genome sequencing

For molecular characterization of PBuV in China, nine positive samples (including serum and feces) from distinct farms located in different geographical areas were chosen forcomplete genome sequenceing. Sequences of two known PBuVs were chosen as references, and overlapping primers targeting nearly the complete genome were designed (Table 1). The PCR products were electrophoresed in a 1% ethidium-bromide-stained agarose gel, and the nucleic acid in the agarose gel was purified and cloned into the pMD18-T vector (Takara, Shiga, Japan). Three positive clones were chosen and sequenced using the Sanger method (BGI, Guangzhou, China). The nucleotide sequences were edited using the Seqman module of DNAStar package (version 7.1.0). Sequence editing, assembly, and comparison were performed to produce final sequences of the viral genomes using BioEdit (Version 7.0.4) and SeqMan program (DNASTAR).

### Homology and phylogenetic analysis

The nucleotide and amino acids sequences of the genomes and predicted open reading frames (ORFs) were compared with those of other bufaviruses available in the GenBank. Alignment was performed using Clustal W, and genetic distances between sequences were determined using the P-distance model. Multiple alignments of the sequences were performed using MEGA6.0 [13]. Phylogenetic trees were built using the maximum-likelihood method based on the general time-reversible model under 1000 bootstraps.

### Nucleotide sequence accession numbers

The PBuV sequences reported here have been deposited in GenBank database under the accession numbers MK279311-MK279319.

## Results

### Brief survey of Chinese PBuVs in China

**Fig. 1:**
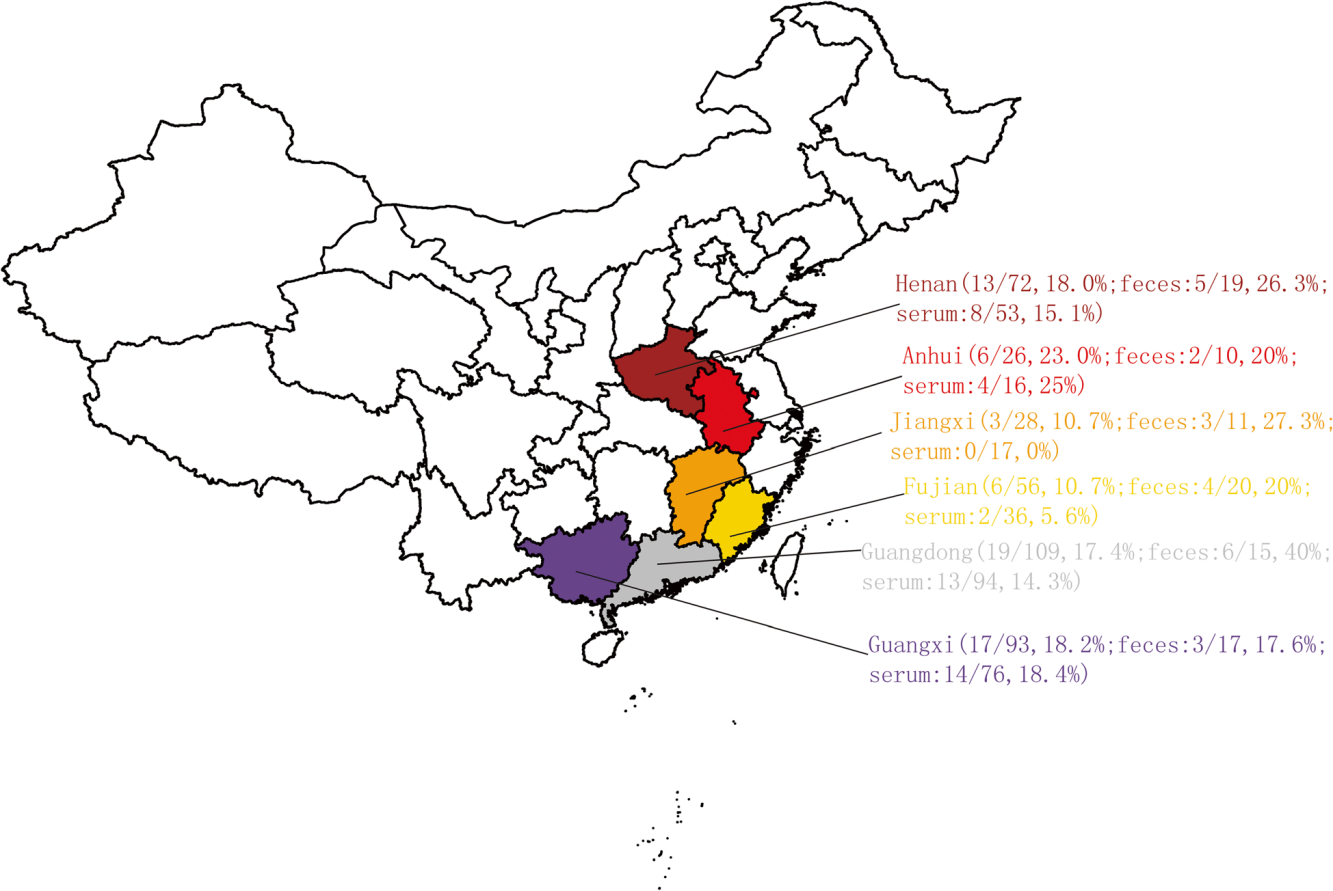
Geographical distribution of PBuVs in China. Six sampling provinces are marked with a unique color with each positive sample/ the total number of samples and the positive rate

In this study, the collected samples were screened using the nested PCR primers, and the amplified target fragments (194 bp) of *NS1* gene were examined by gel electrophoresis. In addition, the expected DNA bands were further confirmed by sequencing. The result of sequencing showed that the nested PCR primers were specific and all the expected DNA bands were identified to be those of PBuVs. The prevalence of PBuV was 16.7% (64/384). Moreover, a higher prevalence rate of PBuV (25%; 23/92 samples) was detected in the fecal samples compared with that in the serum samples (14.0%, 41/292 samples). The PBuVs were distributed in Henan (18.0%, 13/72), Guangdong (17.4%, 19/109), Guangxi (18.2%, 17/93), Fujian (10.7%, 6/56), Anhui (23.0%, 6/26), and Jiangxi (10.7%, 3/28) (Fig. 1).

### Genomic sequencing and analysis of Chinese PBuVs

We successfully determined nine complete genomes of Chinese PBuV from different provinces. These viruses were assembled based on the known Austrian PBuV strain (strain 61; KU867071.1) and Hungarian PBuV Swine/Zsana/2013 (KT96507) [2, 8], which were identified using theviral metagenomics approach. The length of the complete genomes was in the range of 4189–4186 nucleotides (nt), covering four major predicted ORFs: one ORF coding the viral nonstructural protein NS1 (579 amino acids) was nearly 1740 nt and the other ORFs encoding the viral structure proteins—a putative protein (129 amino acids), VP1 (679 amino acids), and VP2 (538 amino acids) —were 2462 nt. In particular, we found the *NS1* gene of one Chinese isolate was 1737 nt. The genetic organization of PBuV resembled that of other bufaviruses from different species [4, 7, 10].

The genetic diversity of the complete genome of Chinese PBuV was 93.3–99.2% and the genome of these Chinese strains showed 94.3–97.8% overall nucleotide identity with the Austrian PBuV (KU867071.1), which was higher than Hungarian PBuV Swine/Zsana/2013 at 91.2–92.3%. Comparative analysis of the near complete gene of other bufaviruses from different species indicated that Chinese PBuV was 57.6–63.1% similar to human bufavirus, 60.9–62.7% to canine bufavirus, 52.8–54% to rat bufavirus, and 58.4–59.5% to bat bufaviruses. Furthermore, the differences in the major ORFs of PBuV were analyzed. The *NS1*, *VP1* and *VP2* genes of Chinese strains shared nucleotide sequence identities of 94.7–99.0%, 92.5–99.4 and 91.2%–99.3%, and shared 94.8–99.3%, 90.5–99.1% and 88.7–99.6% amino acid identity, respectively. In addition, the NS1, VP1, and VP2 nucleotides (amino acid homology) of Chinese strains showed 94.1–97.9% (94.3–99.8%), 90.6–98.7% (88.2–98.5%), and 89.2–97.8% (87.3–99.6%) homology to those of European PBuVs, respectively.

### Phylogenetic analysis of Chinese PBuVs

**Fig. 2:**
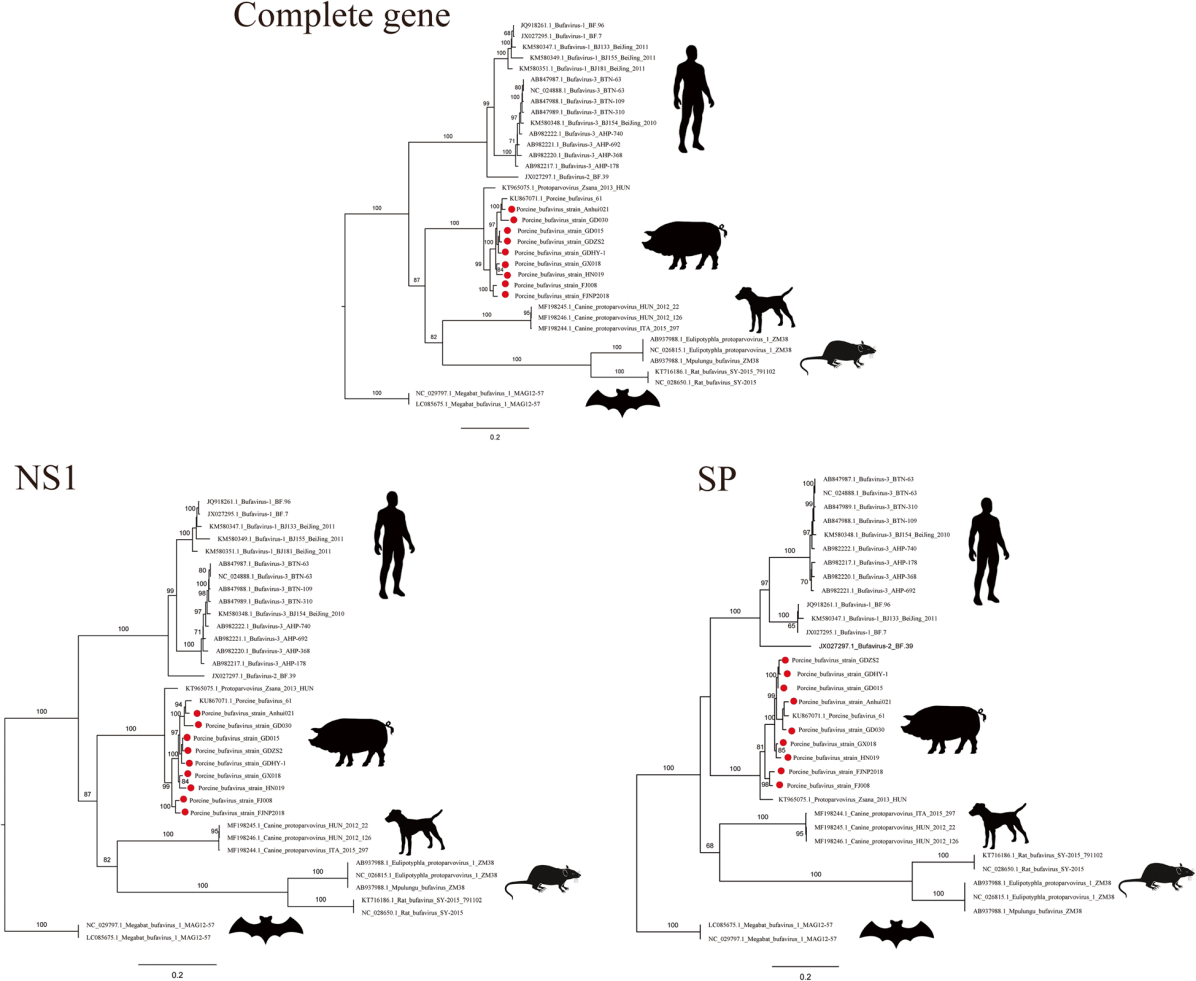
Phylogenetic analysis of PBuV strains in China. The maximum-likelihood method trees were established using Mega (version 6.05) based on the near-complete gene, *NS1* gene, and structural protein (*SP*) gene of the Chinese PBuVs and 27 bufaviruses from different species under the general time-reversible model with 1, 000 bootstraps. The Chinese PBuVs are indicated as *red circles*

Phylogenetic analysis based on the near-complete genome, *NS1* gene, and *SP* gene was conducted. The results revealed that Chinese PBuVs clustered together with known PBuVs. Chinese viruses were closely related to Austrian PBuV strain 61 (KU867071.1). Moreover, these Chinese strains formed four subgroups comprising a host-specific lineage (Fig. 2). Some strains were located in the branch corresponding to its geographic location and the diversity branch of evolution trees also showed the continuous evolution characteristics of PBuVs.

## Discussion

We found that PBuVs are widely distributed in six Chinese provinces. In a previous study, a small-scale epidemiological investigation conducted in Hungary and Austrian revealed a low prevalence of 13.3% [8]. In our study, the large number of samples collected is indicative of more realistic results concerning the prevalence of PBuVs. Viruses were detected in the serum samples; however, the positive detection rate in serum samples was less than that in the fecal samples. It is highly possible that the most prevalent strains in the field are more likely linked to the diarrhea syndrome, indicating the endemic strains may have a strong tropism towards digestive system (e.g., intestine). The high detection rate in fecal sample might result from a local infection in the intestine without antibody responses or the early acute systemic infection. However, with limited knowledge on the pathogenesis of this virus, we can only speculate the reasons underlying this phenomenon and further studies are needed to assess the mechanism of infection and replication of bufavirus.

Our results indicated genetic variability ofChinese PBuV. These results agree with a previous report showing that the evolutionary rate of bufavirus was rapid at 1.6 × 10^− 3^ substitutions per site per year [8]. The large scale and wide distribution of the pig industry in China indicates the higher potential of genetic complexity of Chinese strains. Therefore, we examined the geographic-specific pattern of BuV circulation in this epidemic investigation. This study revealed the molecular characteristics of BuV in China. Considering the lack of knowledge on PBuV, further investigations on epidemiology and the relationship of virus-host coevolution of bufaviruses are needed. According to phylogenetic analysis, Chinese bufavirus was closely related to European strains. With regard to the fact that European countries export pigs and pork products to China, the possibility of Chinese bufavirus originating from Europe should be considered.

To date, the infection mechanisms and pathogenicity of bufavirus have not been well-studied because of the absence of a cell culture system and an animal model. An increasing number of clinical infection cases of bufaviruses have been reported in humans with diarrhea, whereas pathogenicity has rarely been reported in animals [1–6, 9–11, 13, 14]. In a previous epidemiologic study, PBuVs were found to be related to posterior paraplegia in finisher pigs [2]. Although our study indicated that PBuV is widely distributed in Chinese domestic pigs, additional epidemiologic investigations are needed to determine the clinical pathology of the pathogen.

## Conclusion

To the best of our knowledge, this is the first report on PBuV in China, expanding the geographic boundaries of PBuV circulation. The prevalence, complete genomes, genomic characteristics, and genetic diversity of Chinese bufaviruses are presented. These results provide insight into the genetically diverse populations of PBuV circulating in domestic pigs in China and lay a foundation for future studies on bufaviruses.

## Data Availability

All relevant information is provided in this current manuscript.

## Abbreviations

aa: Amino acid
NCBI: National Center for Biotechnology Information
NS1: Non-structural protein 1
nt: nucleotides
ORF: Open reading frame
PBuV: Porcine bufaviruses
PCR: Polymerase chain reaction
VP1: Structural protein 1
wt/vol: Weight/Volume
